# Stress Assessment of Abutment-Free and Three Implant–Abutment Connections Utilizing Various Abutment Materials: A 3D Finite Element Study of Static and Cyclic Static Loading Conditions

**DOI:** 10.3390/jfb16100372

**Published:** 2025-10-02

**Authors:** Maryam H. Mugri, Nandalur Kulashekar Reddy, Mohammed E. Sayed, Khurshid Mattoo, Osama Mohammed Qomari, Mousa Mahmoud Alnaji, Waleed Abdu Mshari, Firas K. Alqarawi, Saad Saleh AlResayes, Raghdah M. Alshaibani

**Affiliations:** 1 Department of Maxillofacial Surgery and Diagnostic Sciences, College of Dentistry, Jazan University, Jazan 45142, Saudi Arabia; dr.mugri@gmail.com; 2 Department of Prosthetic Dental Sciences, College of Dentistry, Jazan University, Jazan 45142, Saudi Arabia; kreddy@jazanu.edu.sa (N.K.R.); drsayed203@gmail.com (M.E.S.); drkamattoo@rediffmail.com (K.M.); 3 Intern Clinic, College of Dentistry, Jazan University, Jazan 45142, Saudi Arabia; osm2545@gmail.com (O.M.Q.); mousaalnajei@gmail.com (M.M.A.); alwaleedabdu111@gmail.com (W.A.M.); 4 Department of Substitutive Dental Sciences, College of Dentistry, Imam Abdulrahman Bin Faisal University, Dammam 31441, Saudi Arabia; fkalqarawi@iau.edu.sa; 5 Department of Prosthetic Dental Sciences, College of Dentistry, King Saud University, Riyadh 12372, Saudi Arabia; salresayes@ksu.edu.sa; 6 Department of Clinical Dental Sciences, College of Dentistry, Princess Nourah Bint Abdulrahman University, Riyadh 11671, Saudi Arabia

**Keywords:** dental implants, dental implant abutment design, dental stress analysis, finite element analysis, dental implant–abutment connection, zirconium oxide

## Abstract

Background: The implant–abutment interface has been thoroughly examined due to its impact on the success of implant healing and longevity. Removing the abutment is advantageous, but it changes the biomechanics of the implant fixture and restoration. This in vitro three-dimensional finite element analytical (FEA) study aims to evaluate the distribution of von Mises stress (VMS) in abutment-free and three additional implant abutment connections composed of various titanium alloys. Materials and methods: A three-dimensional implant-supported single-crown prosthesis model was digitally generated on the mandibular section using a combination of microcomputed tomography imaging (microCT), a computer-assisted designing (CAD) program (SolidWorks), Analysis of Systems (ANSYS), and a 3D digital scan (Visual Computing Lab). Four digital models [A (BioHorizons), B (Straumann AG), C abutment-free (Matrix), and D (TRI)] representing three different functional biomaterials [wrought Ti-6Al-4Va ELI, Roxolid (85% Ti, 15% Zr), and Ti-6Al-4V ELI] were subjected to simulated static/cyclic static loading in axial/oblique directions after being restored with highly translucent monolithic zirconia restoration. The stresses generated on the implant fixture, abutment, crown, screw, cortical, and cancellous bones were measured. Results: The highest VMSs were generated by the abutment-free (Model C, Matrix) implant system on the implant fixture [static (32.36 Mpa), cyclic static (83.34 Mpa)], screw [static (16.85 Mpa), cyclic static (30.33 Mpa), oblique (57.46 Mpa)], and cortical bone [static (26.55), cyclic static (108.99 Mpa), oblique (47.8 Mpa)]. The lowest VMSs in the implant fixture, abutment, screw, and crown were associated with the binary alloy Roxolid [83–87% Ti and 13–17% Zr]. Conclusions: Abutment-free implant systems generate twice the stress on cortical bone than other abutment implant systems while producing the highest stresses on the fixture and screw, therefore demanding further clinical investigations. Roxolid, a binary alloy of titanium and zirconia, showed the least overall stresses in different loadings and directions.

## 1. Introduction

The observation of osseointegration between viable bone and functionally graded biomaterials has led to the development of varied endosteal dental implants that are segregated by materials used, design, types, clinical use, and functions [1]. Endosteal implants have expanded to support various dental prostheses ranging from a single supported crown to a complete denture prosthesis, making them an essential treatment option in prosthodontics [2]. Dental implant interfaces [bone–implant, gingiva–abutment, and implant–abutment] are sensitive contact zones where viable tissues and biomaterials are in direct contact. These interfaces determine implant performance, longevity, success, and patients’ oral health outcomes [3,4]. The bone–implant interface represents a direct bone contact that requires successful osseointegration for implant stability, retention, and functional load transfer. It inevitably represents the healthy interface by showing continuous bone growth with embedded collagen fibers around the implant surface [3]. The gingiva–abutment interface makes contact with the gingiva and serves as a preventive mechanism against bacterial influx, which can potentially lead to peri-implant diseases [4,5]. The implant–abutment interface can take different forms/shapes (flat, conical, tapered, external, internal, spline, Morse, etc.), with each design offering its specific advantages and disadvantages [3,4,6]. The implant–abutment interface has been traditionally classified according to the geometry and technical design of the abutment-to-implant reception as internal, external, or one-piece type, with the latter commonly representing a tissue-level implant type [7]. Based on the geometrical configurations and the type of mechanical retention they impart, each type is further subcategorized to be either a butt-joint, platform-switched, conical-morse taper, or hexagonal connection. The hexagonal connection can be either internal or external. The inefficient sealing of the interface increases the risk of peri-implant diseases and bone loss [2,5,8]. Among various implant designs, external connections have been associated with impaired esthetics, making them less desirable, invasive, and comfortable for the patients [4,9]. External hexagon connections have multiple clinical advantages, like the ease of prosthesis retrieval, an anti-rotation mechanism, and compatibility with technically different implant systems. Internal hexagon connections also enhance restoration stability and resistance with improved force distributions, but at the same time, it may be difficult to adjust divergent implant angles. The taper joint system with a conical seal offers advantages of internal hex connections and better implant–abutment interface sealing but can cause difficulty when release is needed [10,11]. Overall, this crucial interface in an implant restoration is responsible for implant fixture and restoration stability, effective load transmission, the prevention of micro-movements, and a biological seal that protects against microbial leakage, preventing infection and bone loss [3,6]. It also enhances the restoration esthetics and long-term functionality. The type of crown restoration material also determines the mechanical complications associated with this interface, which can include interface misfit, abutment screw loosening, risks of screw and restoration fractures, and the weakness of the supporting framework due to corrosion [6]. Cement-retained abutment–implant interfaces have resulted in inflammation and crestal bone loss due to excess cement [3,8,9].

Biomechanical considerations influencing implant treatment planning include occlusal load (frequency, direction, magnitude), prosthesis material, bone–implant–gingiva-abutment interface (nature, type), bone (quality, quantity), and implant fixture geometry (diameter, length, shape, and thread design) [1,4,6,9,12]. Many factors that influence the outcome of the implant–abutment interface have been investigated. The internal hex implant–abutment system puts more stress on the crestal bone than the tri-channel implant–abutment system and the conical implant–abutment system [10,11]. Better stress distribution has been observed in platform-switched implant systems [13]. Maximal stresses are generally present at the implant neck [1,3,4,5], which increase minimally at the bone–implant interface with increased length, while increased implant fixture diameter produces no significant increase in these stresses [14]. Earlier studies on one-piece implant fixtures indicate that different restorative materials significantly influence stress distribution in the superstructure and the implant [1,9]. Porcelain fused to metal and In-Ceram framework designs transfer less stress to abutments than IPS Empress 2 restorations [15]. Two-piece implants with a lower peak bone strain and peak tooth volume are not suitable for use as small-diameter implants since they tend to produce higher peri-implant bone stresses and strain [16]. Bioactive surface-coated two-piece implant systems (OSSTEM) under all loading conditions have performed better, with the abutment and implant neck both responding favorably, while the system’s weakness is the abutment screw [17]. Shash M. et al. analyzed the impact of various abutment designs on the biomechanical behavior of one-piece zirconia dental implants and surrounding bone tissues, finding the one-piece zirconia implant abutment superior to other abutment designs [18]. Maximum stress in a one-piece abutment body tends to be lower than two-piece abutments with identical lengths and widths (91 Mpa vs. 142 Mpa) [19]. The maximum stress in the last thread of the two-piece abutment screw is also much higher than the body (424 Mpa vs. 142 Mpa) [19]. One- and two-piece narrow-diameter implants both perform adequately under applied loads in both directions; however, it is not advisable to use a narrower one-piece implant for non-axial loads [20]. Axial loading tends to produce similar stress values on different implant–abutment connection systems (external hexagon, internal hexagon, and Morse taper); however, oblique loading increased the stresses generally, with internal connection systems showing lesser stress distribution patterns [10,21]. Different biomaterials have been investigated for use as abutment and implant fixtures to overcome the complications occurring at the implant–abutment interface. With titanium abutments causing esthetic issues in peri-implant tissues, zirconia abutments with various implant–abutment interface geometries and designs gained interest due to their high flexural strength and structural reliability [1,22]. The vulnerability of zirconia abutments to bending, low-temperature degradation, and subcritical crack growth leads to wear on the titanium implant body, which affects the longevity of the implant–abutment interface [8,23]. Fretting wear occurs when titanium transfers to ceramic, causing the damage and loosening of the abutment [24]. Two-piece implant abutments, derived from prefabricated titanium and polymerized zirconia, offer comparable mechanical stability and an increased fracture load compared to one-piece zirconia abutments while achieving less wear at the implant abutment interface [16,22,24]. Additional abutment materials explored include resin–Matrix ceramics and polyether ether ketone (PEEK) [25], which have been observed to be associated with high stress values in the implant components, restorative crowns, and cortical bone. Zirconia implants, with lower stress–strain means, are less likely to cause bone overload and loss in high-stress areas, making them suitable for non-grafted edentulous or rapid extraction [26]. The offset configuration in zirconia implant fixtures should be avoided since it generates higher stress on the bone [27]. Hybrid abutment crowns (monolithic restoration bonded to a titanium base) are more esthetic and cost less comparatively.

Restoration material significantly influences the mechanical stability of implant systems [15,25,28,29]. New high-translucency zirconia materials (5Y-TZP and 4Y-TZP) enable monolithic zirconia crown production, reducing costs and decreasing ceramic thickness. This technology also facilitates faster treatment procedures due to automation, simplicity, and precision, thanks to digital technologies and ceramic materials [29]. The new Matrix dental implant system is made for digital manufacturing methods like CAD/CAM milling or 3D printing, which lets restorations be placed directly on implants without needing extra parts like abutment (abutment-free implant) or manual cement. The Scan & Smile Solution combines Matrix implants with digital manufacturing, allowing for quick placement, scanning, and provisional crown inserting in less than an hour. The system includes two implant systems—multi-level and bone level with eight integrated applications, including Matrix SlimNeck, PowerBase, SmartLock, and SmartBolt [30]. The implant system is available for both immediate and delayed loading, the indication of which depends upon the clinical decision by the dentist. An earlier version of the same manufacturer (TRI) does have an abutment, with both systems using titanium grade 5 ELI (extra low interstitial) (Ti-6Al-4V ELI) alloy [31]. Südbeck S. et al. [32] compared the bending moment of abutment-free (Matrix, tissue-level) implants restored with different materials (3Y-TZP, 5Y-TZP, 4Y-TZP, and CoCrMo) and found higher bending moments in implants without a titanium base for all zirconia restoration groups. FDP fractures were mainly observed for 5Y-TZP. He concluded that, both implant types exhibited similar values after aging, suggesting that implants without a titanium base show equally sufficient stability for clinical applications. In a different study, the conventional cement-retained restoration model showed raised von Mises stress and strain values, making the cementless screw-retained zirconia crown with titanium base abutment a superior restoration alternative [33].

Another significant development for improving implant–abutment interface clinical behavior has been the introduction of binary alloys like Roxolid [83–87% Ti and 13–17% Zr]. Roxolid implants have improved mechanical properties compared to TAV, with a tensile strength of 953 MPa, making them suitable for narrow implants in rehabilitation zones with poor bone width, as opposed to TAV’s 680 MPa strength [34]. Like the TAV alloy, the Ti-15Zr dental implant alloy has a Young’s modulus of 102–104.7 GPa and a Poisson coefficient of 0.33, but it has higher tensile strength [35]. A finite element study found no transmitted stress or deformation magnitude variations between Ti-15Zr and TAV alloys. The Ti-15Zr alloy had a much greater bone-to-implant contact (BIC) percentage than the TAV alloy after 6 weeks of osseointegration [36]. Within the titanium alloy family, wrought titanium–6aluminum–4vanadium ELI (extra-low interstitial) (BioHorizons) [37] alloy and grade 5 ELI (titanium grade 23, Ti-6Al-4V ELI) (Tri) [31] are currently being used to make implant fixtures. The reduced levels of impurities such as oxygen, nitrogen, carbon, and iron compared to regular Ti-6Al-4V improve ductility, fracture toughness, fatigue performance, and biocompatibility [38]. Photoelastic resins, digital image correlation, strain gauges, loss coefficients, and two- and three-dimensional finite element analysis (FEA) have been used to study the transmission of static and dynamic forces to the peri-implant region in vitro due to the difficulties of conducting clinical trials on stress distribution in implant-supported prostheses [3,4,11,13,16,19,21]. FEA is a non-invasive method for assessing material qualities, visualizing layered structures, and identifying pressure sources while maintaining physical attributes and providing stress points for theoretical testing. This in vitro study using 3D FEA was aimed to estimate the stress distribution (normal, shear, principal, and stress intensity) on a single implant-supported prosthesis (mandibular premolar) of four different implant–abutment connections (three with abutment and one abutment-free) using different restorative materials (composite, polytetrafluoroethylene, and zirconia (highly translucent monolithic zirconia)) on implant structures (crown, abutment, fixture, and screw) and surrounding bone (cortical and cancellous). The objective of the study is to determine the material-related stress pattern differences among four implant systems with diverse material characteristics. The null hypothesis would be that there would be no differences between the stresses generated by different implant–abutment interfaces while decreasing the stresses for abutment-free implant systems.

## 2. Materials and Methods

Ethical approval: This study was a part of student research that required due approval from the university ethical committee (HAPO-10-Z-001) through the concerned college. The approval for the study was obtained via reference number REC-46/06/1261, dated 24 December 2024.

Study design: This study followed a comparative experimental tactic with predefined similar independent variables [screw-retained crowns, internal hex, conical/tapered, implant length and width, bone-level implants with minimal collar, implant shape, crown size, and restorative material] and dependent variables (von Mises stresses).

Sample size and simulation runs: Based on the accuracy of the outcomes in terms of the representation of material behavior and stress distribution, four models were designed, each representing a specific implant system. All individual models were run repeatedly with progressively finer meshes until the results were consistent and stable. The simulation runs varied for each model, with the chosen mesh being the one that balanced computational costs and result accuracy. Each simulation run had been set at a confidence interval of 90% and a margin of error of ±3%. Higher confidence levels and smaller margins of error, with less variability, yield precise results that represent the true patterns.

**Figure 1:**
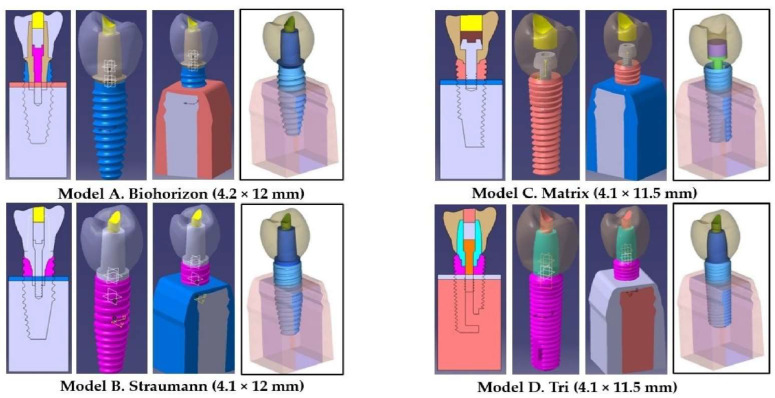
Structural components (fixture, abutment, screw, crown, cortical, and cancellous bone) of each model specified for making up the finite element analysis model (figure created using MS Paint, version 20H2 (OS build 19042,1466), MS PowerPoint, windows 10 Pro, Microsoft corporation). [The implants were placed 3 mm supracrestally in order to replicate the “worst-case conditions” as prescribed by the ISO 14801:2016(E) for testing, making the implant appear supracrestal].

The generation of the individual implant components and whole implant models (Figure 1): The study used a 3D digital model to simulate prosthodontic rehabilitation and assess stress (von Mises stress) on the crown, abutment neck, abutment screw, and cortical/cancellous bone of a mandibular first premolar. Microcomputed tomographic imaging (microCT, Quantum GX; PerkinElmer, Shelton, CT, USA) and SolidWorks (Dassault System, Waltham, MA, USA) were used to build a stereolithography file from an undamaged mandibular first premolar. Using flexible pixel densities and a three-point cloud, a medical interactive image control system detached scanned dental data into diverse types of alveolar bone (compact and cancellous). After image segmentation and spline reconstruction using the STP format, the adult mandible’s geometry was modeled after the shape generated from the CT database. The mandibular first premolar area was modeled based on findings from Shemtov K. et al. [39]. Since NURBS (Non-Uniform Rational B-Splines) are mathematical representations of three-dimensional geometry, they recreated the implant components and surrounding structures’ geometrical shapes. The essential shapes are accurate and consist of specifically detailed mesh or point cloud derivatives. The CAD program (SolidWorks 2014, Dassault Systèmes) then rebuilt the model in 3D. Each implant system model was designed by the integration of independently industrialized models from real element components for each specific implant system. The implant component geometry was accurately measured using a 3D digital scan (Visual Computing Lab, Pisa, Italy) and translated into an STL mesh. The specifications of each implant system are presented in Table 1, while the integrated components for each implant system and model are shown in Figure 1. Model A was representative of the BioHorizons Implant System (Hoover, AL, USA) [37] (Figure 1 Model A); Model B represented the Straumann (Institut Straumann AG, Basel, Switzerland) implant system (Figure 1 Model B) [40]; Model C represented the abutment-free Matrix (TRI Dental Implants, AG, Hünenberg, Switzerland) implant system (Figure 1 Model C) [30]; and Model D represented the implant system with the abutment of the same manufacturer (TRI Dental Implants, AG, Switzerland) (Figure 1 Model D) [31]. The length and width taken for each implant were decided on the basis of their commercial availability, which meant that all implant systems were 4.1 mm wide except for BioHorizons, which was 4.2 mm, and two implant systems (BioHorizons and Straumann) were 12 mm long, while the other two were 11.5 mm long. The material used for each implant system and their related specifications are shown in Table 1. For obtaining a solid crown model, a high-resolution three-dimensional exocad model was introduced to the CAD program while creating the geometric shape of the premolar crown for each implant system according to the dimensions of the abutment. The mandibular second premolar crown was digitally constructed using anatomical data which conformed to the outer dimensions for each respective abutment of the three implant systems used [41]. Sequential software processing was performed so that the three-dimensional crown model would be based upon the geometric dimensions. The surface contours and meshes of the second premolar crown were then entered into the SolidWorks (Dassault System, Waltham, MA, USA) and the intact solid second mandibular premolar model was thus generated with dimensions that matched the natural crown [length 7.5 mm, width 7 mm, mesiodistal diameter 7 mm]. For the Matrix implant system, the solid model did not have any abutment; therefore, an entire block of the crown was digitally designed. The Matrix implant abutment connection has a 20 degree internal flat connection and a 20 degree shoulder. The crown length represented the abutment length for this implant system. The Matrix implant receives its retention through the vertical rotation blockers and a frictional precision fit between the implant and the base. Platform-switched Matrix implants also enhance retention and stability. Anti-rotation for the Matrix implant system is achieved by the internal hexagons and anti-rotation-milled titanium base. The Matrix abutment screw was used with a 2.6 mm diameter and a flat horizontal head. The average volume of the crown was calculated to be 10.8 mm^3^. Except for the Matrix implant system, the framework core accounted for a higher proportion than the overlying crown restoration. A crown restoration made from highly translucent monolithic zirconia was used to distribute loading, after the closure of the occlusal screw hole space with composite resin restoration, simulating the clinical protocol.

An ideal relationship (100% osseointegration) between implant and bone was assumed for total osseointegration at the bone–implant interface. With this condition, a perfect bond between bone and implant is assumed with zero relative motion in any direction, thus simplifying the model and representing the ideal integration of the bone to the implant fixture. The simulation of the fixed interface was performed under Dirichlet boundary conditions while the applied load or forces on the implant and surrounding tissues were simulated using Neumann boundary conditions. Both boundary conditions correctly model the implant’s mechanical environment, thus ensuring accurate simulation results. The mandibular base was considered a fixed border, while its planes were frictionless, thus signifying it as a normal restriction. Thus, the base of the mandible constrained the degree of freedom of nodes in three directions. In addition, these conditions were also applied to anatomical structures that were distant from the implant, where relative movements are absent. The FEA models were validated against published data by comparing the FEA predictions to previously published reference data, using digital image correlation (DIC), in which strain and displacement fields were compared quantitatively. The DIC-leveling processed the FEA data by simulating it in a DIC engine while matching the spatial resolutions and accordingly filtering experimental data. This allowed quantitative error mapping between the two simulated and experimental fields to overcome the limitations of direct interpolation. Direct interpolation can cause false validation results due to differing spatial resolutions. Different individual FEA implant components were also aligned against images using pressure distributions after spatial transformations and the data alignment of sets. Differences were then quantitatively corrected using error metrics like root mean square errors to assess the prediction accuracy of pressures caused during contacts.

Mesh definition and convergence: To achieve accuracy and reliability using the finite element method, specifying the mesh size and the type of elements is significant. Smaller elements were used that improved the result precision and accuracy. Finally, finite element analysis software (ANSYS version 10; ANSYS Inc., Canonsburg, PA, USA) was used to import the geometries from four separate models in STEP format. The software went a step further by breaking down the geometric designs into several meshes made of nodes and tetrahedral elements. The use of a mesh allows for the discretization of a continuous geometric space into pieces that are mathematically defined and reliably formed. This discretization allows computers to numerically solve the governing equations and mimic the physical effects. At each node, the element has freedom in three different directions relative to the global coordinate system: x, y, and z. A stable solution independent of the mesh size was achieved, particularly in the impact zone, by a refinement procedure that was carried out during the mesh creation process, guaranteeing great accuracy in this area. The mesh grid reported relative errors for the maximum von Mises stress in the implant system and surrounding bone, calculated as the percent difference between current stress measurements and earlier trial run predictions. Convergent calculation and mesh grid acceptance were determined when relative errors were fewer than 1%. Due to the elevated accuracy of the nodal solution, it was needless to employ an area to attain a regular solution after this refinement. As a result, the matching mesh was deemed ideal. A high-quality mesh has its own consequence on simulating speeds, convergence, and their relative accuracy on the analysis. As the numerical model parameters, like mesh density or iteration steps, are adjusted, convergence occurs, which is a steady state of the computed solution with low change. This is a crucial part of finite element analysis (FEA). A 10% mesh control, a growth rate between 1.2 and 1.5, and an edge length no greater than or equal to one-fifth of the smallest structure’s circumference were all used to run the test. The number of nodes or elements required to construct the models was determined by the outcomes. Table 2 presents the precise nodes and element counts used for each component in this FEA investigation. Ansys software regulates the aspect ratio, ensuring that the precision is relative to mesh size. Finer meshes yield more precise resolutions, whereas coarser meshes are required in closeness to loading points and threaded components. The findings of finite element analysis can be evaluated independently for each component or combination of different components in a group. Since one of the implant systems (Matrix) did not have an abutment, it was therefore considered necessary that any additional analysis in the form of combined crown and abutment in all implant systems allows comparisons of VMSs.

Material properties: Table 3 specifies the properties of each material employed during the analysis of the stress distribution of four different implant models in this study. The program assumed consistent homogeneity, isotropy, and linear elasticity in simulated structures, ensuring uniformity in all directions and independence from strain rate. Therefore, the materials modeled were linear elastic, isotropic, and homogenous. The mechanical properties are outlined according to the manufacturers’ declarations.

Masticatory loading condition: Mechanical testing based on the ISO 14801 standard is generally used to evaluate the performance of the dental implant system according to material and design changes [42]. Three loading conditions were considered that included static axial, static oblique, and cyclic static, thus fulfilling the objectives of loading types (static axial, cyclic static) and direction (axial, oblique) [16,20,40]. The loading was performed for individual components (implant fixture, abutment, crown, screw, restoration (cement), cortical/cancellous bone) first, followed by combined (abutment/crown, whole model, cortical and cancellous bone) components. During the simulation of the osseointegration process, two dissimilar models (frictional and bonded) represent different integration properties at the junction of the implant with bone tissue. As an example of the quality of integration, the friction coefficient between the abutment, implant, and screw interface was 0.5, and 0.4 between the implant, cortical bone, and cancellous bone interface, according to the contact type of frictional analysis [43]. The friction coefficient, typically 0.5, affects the screw preload and stability in the implant–abutment–screw complex, affecting torque translation into preload. Higher friction reduces efficiency and increases screw loosening [43]. On occlusal force application, a frictional contact denoted the existence of a gap between the implant and bone, while nonmoving contact surfaces were considered bonded. The three-dimensional loading of the implants with lingual (17.1 N), axial (114.6 N), and mesiodistal (23.4 N) forces, respectively, representing the average oblique (75 degrees to the occlusal plane) force of chewing in a natural setting [21,42,43]. At this predefined angle, these slices of the crown developed a masticatory force of 118.2 N [44]. The buccal cusp’s lingual inclination was influenced by this three-dimensional loading. A time-dependent masticatory load (Fdynamic) was then used for the cyclic static analysis [44]. These calculations were predicated on the idea that a person chews for three periods every day, with each session lasting fifteen minutes and a chewing pace of sixty cycles per minute (1 Hz). That works out to approximately 985,000 chewing cycles annually, or 2700 chewing cycles daily [45], with typically 800 to 1400 chewing cycles for meals constituting normal diet and another 1200 to 1600 cycles for non-chewing functional movements like swallowing.

VMS measurement and statistical interpretation: The findings were characterized as corresponding to von Mises stress, which, when operationally defined, refers to a numerical value, that, when applied to the three-dimensional primary stresses, yields an operative complete magnitude of stresses. The assessment of the four models was performed by the finite element processor, which, after processing, displays color-coded plotted results. Colors are divided into different bands, with each color band denoting a range of stress levels. As an indicator, the color ‘red’ indicates the maximum von Mises stress levels, which are depicted on the area of the structure, while ‘blue’ represents the least VM stress levels. VMS levels were measured for implant fixture, screw, cortical bone, cancellous bone, individual abutment and crown, and finally abutment and crown together. Since one of the models (Model C) was abutment-free, the impact of eliminating the abutment was judged by measuring the abutment and crown together for all models.

## 3. Results

**Figure 2:**
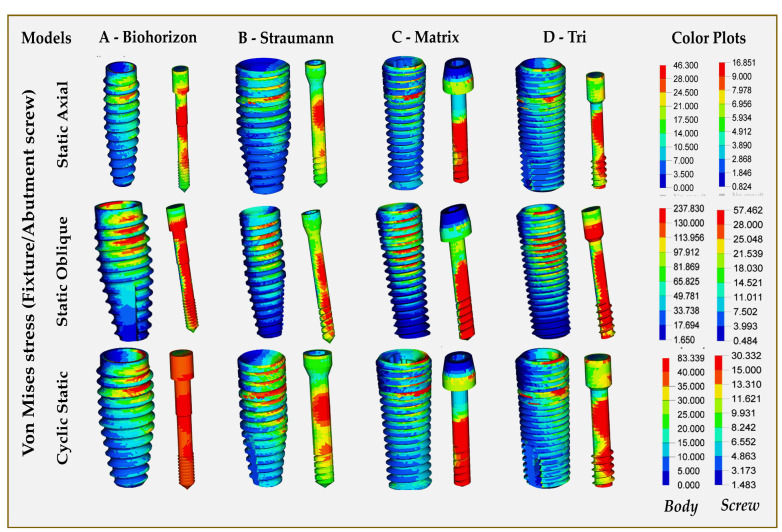
Finite element analysis showing von Mises stress distributions on implant fixture and abutment screw in different implant–abutment interface models.

Implant fixture and abutment screw: Figure 2 displays the stress distribution levels in implant fixtures subjected to both static and cyclic static loading for all models. The maximum VMS for implant fixture in static and cyclic static loads was seen in Model C (Matrix, abutment-free, titanium grade 5 ELI), while model D (Tri, with abutment, titanium grade 5 ELI) showed the highest stresses in oblique loading (237.83 MPa) (Table 4). Model B (Straumann, Roxolid) exhibited the lowest stress levels on the implant fixture across all loads. Observed stresses were located in the superior threads for all implants under all loads, with Model B showing the higher distribution of stresses between threads. Under all loading scenarios, the maximum stress values at the implant body were below the material yield strength (462 MPa). For all loadings, the highest VMS was observed in Model C (Matrix, abutment-free, titanium grade 5 ELI), with higher stresses concentrated in the lower third part of the screw (Figure 2). Higher stresses throughout the length of the abutment screw were observed for Model A under static and cyclic static loading. The lowest stresses in the abutment screw for all loading conditions were seen in Model B (Straumann, Roxolid).

**Figure 3:**
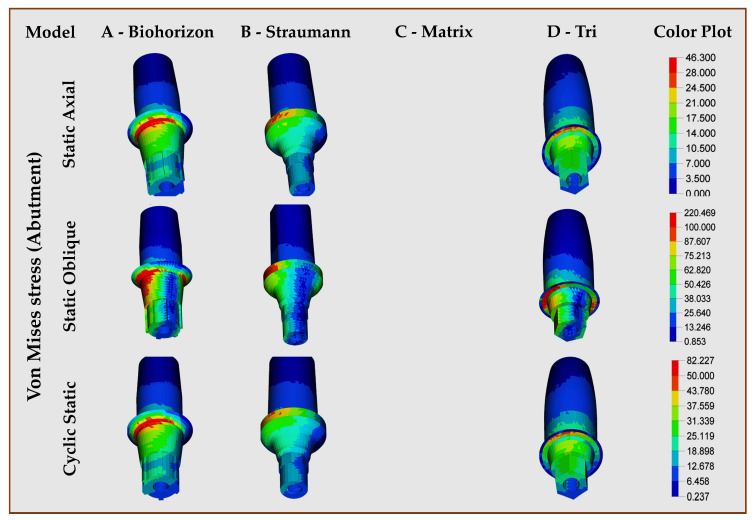
Finite element analysis showing von Mises stress distributions on implant abutment in different implant–abutment interface models.

**Figure 4:**
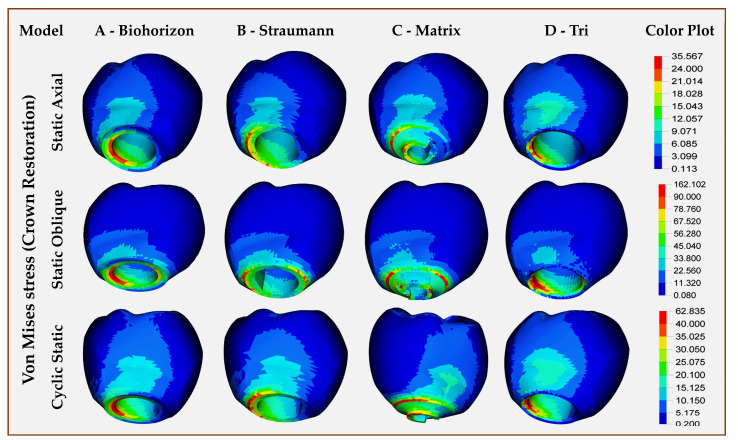
Finite element analysis showing von Mises stress distributions on individual monolithic zirconia single-crown restorations in different implant–abutment interface models.

**Figure 5:**
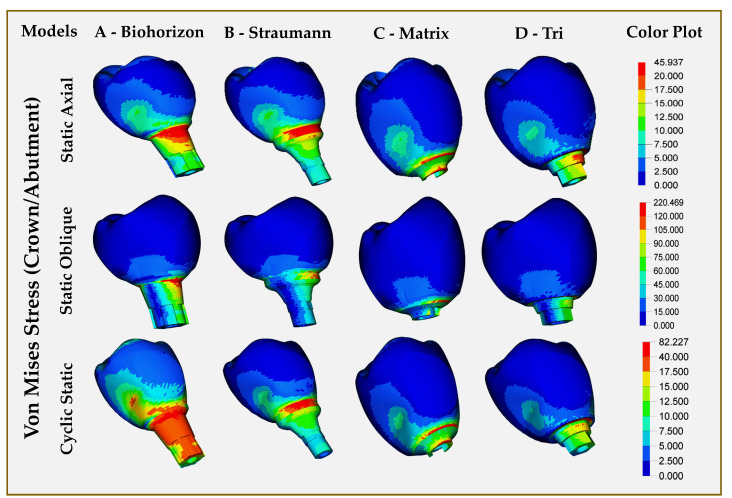
Finite-element analysis showing von Mises stress distributions on individual monolithic zirconia single-crown restorations and abutment in different implant–abutment interface models.

Abutment, crown (individual, combined): Out of the three models with abutments, the highest abutment stresses were in Model A (BioHorizons, wrought titanium) in both directions, axial/oblique and static/cyclic static loadings. Model B (Straumann, Roxolid) showed almost twice fewer stresses than Model A in static and cyclic static loadings. For all abutments, the stresses were concentrated in the middle portion, which had a maximum diameter (Figure 3). Figure 4 presents the stresses within the crown restorations of each model. Model D crown (Tri, with abutment, titanium grade 5 ELI) had the highest VMSs in both static (35.57 Mpa) and cyclic static (62.84 Mpa) axial loading, while Model C crown (Matrix, abutment-free, titanium grade 5 ELI) had the highest stresses in oblique (138.44 Mpa) loading. The FEA of the crown with abutment showed the highest VMS levels in all types of loading for Model A (BioHorizons, wrought titanium), while Model B (Straumann, Roxolid) showed the least stress values for both static and cyclic static, axial and oblique loads. The VMS of Model C (Matrix, abutment-free, titanium grade 5 ELI) was lower than model D (Tri, with abutment, titanium grade 5 ELI) and Model A in static and cyclic static loads. Under oblique loading, however, the abutment-free had higher stress levels (214.05 MPa), which were almost similar to Model A (Table 4, Figure 5). For all models, the concentration of higher stresses was at the cervical portion of the crown (Figure 5).

**Figure 6:**
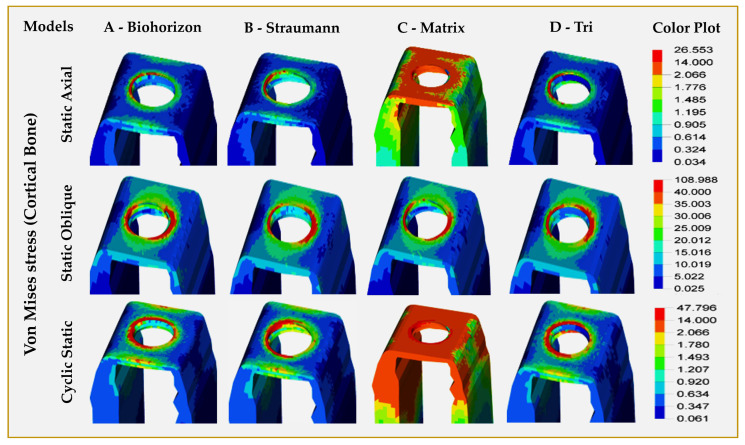
Finite element analysis showing von Mises stress distributions on the cortical bone surrounding dental implants in different implant–abutment interface models.

**Figure 7:**
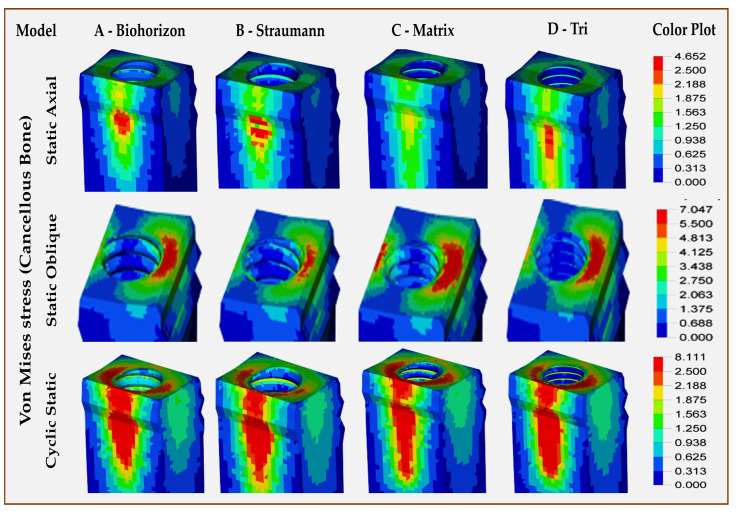
Finite element analysis showing von Mises stress distributions on the cancellous bone around the zirconia single-crown restorations in different implant–abutment interface models.

Cortical and cancellous bone—Table 4 (Figure 6 and Figure 7): Model C (Matrix, abutment-free, titanium grade 5 ELI) showed the highest VMS in cortical (compact) bone for all types of loading, with the highest stress levels for oblique loading, which are almost twice the values observed in other 3 models. The lowest stress levels in cortical bone were observed for Model B (Straumann, Roxolid) for both static and cyclic static loading in either axial or oblique directions. In all models, the stress patterns in the compact bone were concentrated around the implant fixture neck for all types of loading (Figure 6). The difference between abutment-free and models with abutments was the spread of stress in abutment-free (Model C), which not only involved the horizontal part of the mandible but also spread across the vertical cortical plates (buccal and lingual), especially during cyclic static loading (Figure 6). There were wide variations for stress levels observed in cancellous bone among different models under different loads. The highest static axial-related stress levels in cancellous bone were observed in Model B (Straumann, Roxolid) (Figure 7), while the highest static oblique-related stress levels were seen in Model C (Matrix, abutment-free, titanium grade 5 ELI). For cyclic static axial-related stress levels, the highest values were seen for Model B (Straumann, Roxolid) and the lowest were seen for model D (Tri, with abutment, titanium grade 5 ELI).

General clinical application inferences: The results indicate that some patterns can be generalized: oblique loads generate more stresses than axial loads, and cyclic static loading produces higher loads than static loading. Implant fixture composition influences the stresses generated in various implant components. Roxolid [83–87% Ti and 13–17% Zr] was associated with less stress generation in all components except cancellous bone, while wrought titanium alloy was associated with high stress generation in most of the implant components. Eliminating abutment increases stresses in implant fixture, screw, and cortical bone. The absence of abutments doubles the stresses on the cortical bone, which are spread over the buccal and lingual cortical plates.

## 4. Discussion

This study estimated the stress level distributions to various implant components and adjacent bone of four different implant–abutment connections. One of the implant systems has recently been introduced, which has eliminated the abutment completely, and therefore demands investigation. The four implant systems chosen in this study simultaneously understand the role of different implant fixture materials, while one of the implant systems, ‘Tri,’ is from the same company that has introduced abutment-free implants, with ‘Tri’ being the version in which abutment exists. These four different implant systems allow the analysis of three different implant biomaterials (wrought titanium-6 aluminum-4 vanadium ELI alloy, Roxolid, and titanium grade 5 ELI), besides also allowing a comparison with the abutment version of the same manufacturer (TRI). The main findings of the study were that the abutment-free model was associated with the highest stresses on the implant fixture, screw, and cortical bone under all types of loading; this produced the highest stress on the crown and cancellous bone under oblique static loading; and the wrought titanium alloy used in BioHorizons implants produced the highest stress levels under both static and cyclic static loading on the individual abutment and abutment-with-crown combination. Overall, the abutment-free implant generated more stresses under oblique loading. The implant system that used a combination of titanium alloy and zirconia produced the lowest stresses under both static/cyclic static and axial/oblique loading for most of the implant components, including both cortical and cancellous bone. Von Mises stress is a significant measuring tool to assess when a ductile material (like most metals) will start to yield or permanently deform under different kinds of stress. It utilizes clinically relevant applied stress directions on a point, combining them into one scalar value, called equivalent stress. The criterion for VMS indicates that yielding will occur once cumulative stress surpasses critical material-specific value, making it highly valuable for assessing failure in engineering design and finite element analysis. Moreover, in the context of this research, VMS, because of its representation of tensile, compressive, and shear stresses together, would offer a more accurate prediction of the material behavior seen in the oral cavity. The implant–abutment interface is significantly strategic in choosing an implant system since it is sensitive and determines the probability of developing biological or mechanical complications [9,24]. A consideration of the interface design and material characteristics is crucial when selecting an implant system [3,4,6,8]. Model C in this study represented the recently introduced Matrix abutment-free implant system. Matrix offers two implant systems: multi-level and bone-level, with platform 3.7 mm (P37) and platform 4.5 mm (P45) for each [30]. Multi-level implants have four different widths, with each having six different lengths, while bone-level implants have three widths and the same lengths. Both are made from titanium grade 5 ELI alloy. The Matrix implant has a self-locking (SmartLock) system for the automatic positioning of crowns and multi-unit restorations, featuring two large vertical rotation blockers for easy milling and tactile feedback [30,31]. The Matrix SmartBolt features three flat horizontal screw heads, designed for material-specific milling strategies, with a specially treated surface for increased hardness, scratch resistance, and fatigue strength, and a narrow screw head for metal restorations. The Matrix implant system offers faster workflow, increased precision by 73%, and significant cost savings. It eliminates abutment, cement, model, and analog limitations, and allows for a 100% screw-retained restoration. The system also features a digital healing collar library for 48 individual components [30]. Titanium grade 5 ELI is one of the best choices for implant alloys due to its high mechanical strength [tensile strength (860–900 MPa), yield strength (795–830 MPa)], superior fatigue and corrosion resistance, biocompatibility, and osseointegration [35,37]. The high VMSs observed in this study may be related to the higher bending moments associated with directly screwed Matrix implant systems reported in a previous study [32]. Higher von Mises stresses in titanium grade 5 ELI (Ti-6Al-4V ELI) indicate a higher likelihood of plastic deformation or material failure under loading conditions [38,46]. The higher stress patterns in various implant components by the Matrix implant system were generated above the implant connection, which substantiates the findings by Hjerppe J. et al. [47], who observed a similar stress pattern in 75 percent of abutment-free zirconia. Internally connected all-ceramic abutments have been reported to be more vulnerable to fractures when compared with metallic abutments [48]. However, since the failure load of implant abutments is much higher than the maximal bite force (446 N) in healthy adults [17,21], with ideal thickness, they are able to survive without failure. Cyclic loading may lead to a lower critical stress limit, resulting in fatigue failure rather than a fracture failure, which is better understood by stress versus the number of cycles to failure (S-N curve), which links stress amplitude to loading cycles prior to failure. Static loading results in an instant fracture, while cyclic loading causes fatigue failure but over a period of several cycles at reduced stresses. S-N fatigue curves found in titanium and its related alloys show decreased fatigue strength with increasing cycle numbers. The fatigue failure results from cracks that tend to be initiated and spread at the implant–abutment contact or, as a matter of fact, any other stress concentration area that has been subjected to cyclic stress. At lower loads than the materials’ static yield limits, slower deterioration can result in catastrophic failure or fracture; hence, implant performance under clinical cyclic occlusal loading tends not to be adequately represented by static stress FEA. The S–N curve of titanium or titanium alloy implants demonstrates significant fatigue limits in biomechanical evaluations, explaining why critical cyclic stress levels are lower than static yield stress and the need to evaluate implant designs and materials to reduce fatigue risk.

Since this study was comparative in nature, the smallest VMSs were associated with Model B, which utilizes binary alloy Roxolid (85% titanium, 15% zirconium) for making the implants. Roxolid implants offer more treatment options than titanium implants due to their apically tapered design, allowing for underpreparation and high primary stability in soft bone. SLActive accelerates osseointegration, providing a safer, faster treatment in 3–4 weeks, reduced healing times, and increased treatment predictability [40]. Our results are in support of the results observed by Brizuela-Velasco A. et al. [34], who reported a lower VMS in Roxolid (89.19 MPa) than in Ti-6Al-4V. He attributed less stress generation in Roxolid to its significantly higher yield strength (953 MPa) and fatigue strength (45% higher) than other titanium alloys. Our analysis of stress distribution to the cortical and cancellous bone also falls in line with his stress distribution results when comparing binary alloy implants with titanium grade 5 ELI [34,35]. Yang F. et al. [49] examined the impact of varying modulus of elasticity (MOE) values on deformation and von Mises stress distribution in dental implant systems (BLT, Straumann, Switzerland) and peri-implant bone tissues under cyclic static cyclic loading. The Zr implant system showed the lowest maximum deformation and von Mises stress in cortical bone, while the PEEK system had the highest maximum von Mises stress. Adjusting the MOE can modify the biomechanical characteristics of the implant system [15,25,28]. After dental implant placement within the viable bone, the remodeling of bone takes place around the implant surface, which includes selective bone resorption and deposition. The mechanical stimulation of the implant is crucial to reduce peri-implant resorption, which plays a significant role in controlling the overall bone volume. The balance between the bone deposition and resorption is critically maintained through mechanical stimulation within physiologic limits. Since mechanical stimulation occurs within the implant fixture, the amount of bone deposition or bone mass is highest near the implant while declining with distance. The stability of the implant increases as bone deposition occurs around the implant, followed by the calcification of the newly deposited bone. Other significant factors influencing bone gain are the optimal magnitude of the strain within the bone and the loading frequency. Osteoblasts under appropriate mechanical stimulus accelerate the deposition of the bone matrix around the implant fixture. Restorative material properties are thus essential to ensure that optimal stress on the bone–implant interface is imposed to enhance bone deposition that is ahead of bone resorption.

Each implant system works on its own customized biomechanics and, relative to the design, generates stresses within implant components and surrounding structures. Internal hex connection implant systems generate higher implant–abutment interface stress, while conical or tapered systems provide favorable stresses [13]. However, material and restoration properties simultaneously affect stress generations irrespective of the design considerations [50]. The BioHorizons implant system in our study was associated with relatively higher stresses despite having a tapered internal connection, which is related to the implant material used [wrought Ti-6Al-4Va ELI]. Other implant–abutment connection types, like the tri-channel connection, have intermediate stress levels. Platform-switched implants reduce stress on the crestal bone but can increase stress on the abutment and implant–abutment screw, especially under non-axial loading, which increases stress across all connection types [13,50]. Biomechanics also differ according to the implant type between cement-retained and screw-retained implant systems. In cement-retained implants, the abutment design, type of cement, and its thickness affect the implant biomechanics by providing even stress distributions at implant interfaces, thus minimizing the mechanical risks like screw loosening or fracture since cements are elastic in nature. While cement-retained implants result In reduced stress peaks on alveolar bone, excess cement potentiates greater risk to other mechanical and biological failures [51]. The mechanical retention of screws in screw-retained implant systems depends on the preload for its success. Mechanical failures related to screws can occur if sufficient preload is not generated. Prosthesis fit, torque application, and loading protocol are also critical to desirable implant biomechanical behavior. Lateral forces increase implant–abutment interface overloads, while axial loads decrease screw-associated strain. Biomechanically, the abutment-free implant system eliminates microgaps, thereby eliminating micromovements at the implant–abutment interface, which is a natural site of mechanical complications. The same effect was observed in Model C (abutment-free), where stresses were less in the abutment crown complex in different loading directions. Improved load transmission and stress distribution take place as stress concentration at the abutment joint is removed. While abutment elimination limits prosthetic flexibility, it offers biomechanical advantages like stability, fit, retrievability, and less component failure risk. The biomechanical advantage in abutment-free systems relies substantially on the direct implant-to-prosthesis interface. The connection height and the taper determine that the stress distribution patterns for surrounding structures and the implant fixture. Specialized screws used in such systems have significantly higher compressive and fatigue strengths while showing a low VMS at the abutment–screw interface, as observed in our study [52]. Geometrical differences in the screw and increased diameter permit wider stress distributions, thus lowering the stress at the screw interface with low chances of screw mechanical failures [52]. While these biomechanical advantages are formulated on engineering principles, the long-term consequences and survivability of abutment-free implants still need to be determined through clinical studies.

Higher stress values for all implant systems were observed at the implant’s neck, which is in agreement with previous studies [3,4,6,7,8,9,15,43]. Stress will only be transferred to the initial point of contact between two materials with distinct elastic behaviors (such as Ti and bone) when they are placed in interaction and subjected to load [34]. Lower stress generation in binary alloys has also been attributed to the Ti-15Zr implant showing a more elastic behavior towards load [34]. Stress patterns generated on the bone are also influenced by other implant design features like abutment angulation [16,19,20,53], implant depth in relation to the bone [5,48,54], bone quality [5,9,15,24], thread patterns [55], loading directions [17,21,25,41], and the type of abutment [cement versus screw retained] [3,10,11,13,15,16,19,20,28]. Our results showed that cancellous bone experienced lesser stresses by all models, which is in agreement with a previous study [56]. Our study results demonstrated that Model A [wrought Ti-6Al-4Va ELI alloy] demonstrated the highest stresses on abutment and abutment/crown combined in both static/cyclic static and axial/oblique loads, while producing the second-highest stress levels on implant fixture, screw, cortical, and cancellous bone. Wrought titanium-6Al-4V ELI (extra-low interstitial) alloy is a high-purity version of Ti-6Al-4V commonly used for medical implants due to its excellent strength-to-weight ratio, corrosion resistance, ductility, and fracture toughness. The alloy differs from conventional alloys in that its yield strength and elongation are lower while its ultimate strength is higher than that conventional titanium alloy [57]. Ti-6Al-4V ELI is an alpha-beta alloy with a multiphase structure, causing heterogeneous stress distribution during loading [57,58]. Its presence in the alloying elements in combination with aluminum and vanadium leads to anisotropic mechanical behavior, thereby affecting stress distribution [57,59]. Under stress, Ti-6Al-4V ELI displays complex dislocation activity, leading to uneven stress accommodation and localized deformation [60]. Binary alloys, with fewer alloying elements, have clearer slip systems and more predictable deformation paths [57,58]. The ELI grade is designed for higher ductility and fracture toughness but may sacrifice uniform strength and hardness [60]. According to Bayata F. et al. [61], Ti-6Al-4V implants exhibit reduced stress distribution with an increased taper angle, and improved tightening torques enhance resistance to cyclic biting loadings [62]. With an increased thread number, they offer a long service life, high fracture resistance, and low stress concentration [63,64].

Strengths and limitations: This study investigates a recently introduced abutment-free implant system that has the potential to revolutionize implant dentistry. The study used 3D FEA since two-dimensional models are less efficient and accurate. Three-dimensional FEA visualizes implant stresses; however, dental literature has fewer cyclic static FEA investigations compared to static loads. Few 2D or 3D FEA research models have studied cyclic static forces under impact loads. FEA models have limitations, including potential data inaccuracies, skewed conclusions, and reliance on participants’ computer skills. Several assumptions concerning the bone–implant interface, boundary conditions, material qualities, and model geometry greatly affect the predictive accuracy of finite element analysis (FEA) models. Therefore, the results of this study are only applicable to the conditions and the materials that have been described in the methodology. The assumptions about various constraints and those involving the external forces are simplified due to which there will be differences among patients whose mandibular to maxillary bone relation varies like in class 2 and class 3 skeletal malocclusions. The study is also limited by not having estimated the fatigue failure after cyclic loading due to the financial and time limitations, which will be considered in future studies.

## 5. Conclusions

This study, within the scope of its methodology and limitations related to 3D FEA, concludes that, among various implant systems and their respective differences in material compositions, the binary alloy-based Roxolid (85% titanium, 15% zirconium) (BLT) with a diameter of 4.1 and a length of 12 mm, produced the least stresses on the implant fixture, abutment, and abutment screw, abutment and crown (combined), and cortical bone. The recently introduced abutment-free implant based on titanium alloy (Ti-6Al-4V ELI) produced less stress on static and axial loading as compared to cyclic static and oblique loading. Although the abutment-free implant system was associated with the highest stress generation in some implant components, they may be considered acceptable, while the stresses generated on the compact bone may need further investigations that are clinically designed. This becomes essential since the cost of an abutment-free single implant (USD 250 to USD 350) has been kept higher than conventional implants (100 to 200 USD). This study recommends clinical trials that investigate the short- and long-term effects of abutment-free implants to assure limitations associated with such systems.

## Figures and Tables

**Table 1:** Materials and implant systems (Table created using MS Word, version 20H2 (OS build 19042,1466), windows 11 Pro, Microsoft corporation)).

**System**	**Manufacturer**	**Features**
BioHorizons (TPC) (Model A)	BioHorizons Implant Systems, Inc., Birmingham, AL, USA.	Class II (21 CFR 872.3640)—screw-type endosseous dental implant. Types: tapered pro [width—3.4, 3.8, 4.6 mm; length—9, 10.5, 12, 15, 18 mm] and tapered pro conical [width—3.3, 3.8, 4.2, 4.6, 5.2 mm; length—9, 10.5, 12, 15, 18 mm; 7.5° conical connection]. Material: wrought titanium–6aluminum–4vanadium ELI alloy (UNS R56401) [yield strength 795–860 Mpa, UTS—860–970 MPa, elongation—10–15%, MOE—approximately 105–115 GPa]. Features: Laser Lok (1.8 mm) microchannels, resorbable blast texture media (tricalcium phosphate). Abutments: custom and CAD/CAM abutments [hybrid base abutments, Laser-Lok titanium-based abutments, multi-unit abutments (straight and angled—17–30 degrees)].
Straumann (BLT) (Model B)	Institut Straumann AG Peter Merian-Weg, Basel, Switzerland	Types: soft tissue level tapered, bone level tapered. Material: Roxolid (85% titanium, 15% zirconium). Design features: CrossFit connection, bone control design, consistent emergence profiles. BLT implant: width—2.9, 3.3, 4.1, 4.8 mm; length—8, 10, 12, 14, 16, 18 mm. Properties: implant [strength—850 Mpa, elongation −12%, MOE −98 GPa], Abutment [strength—900 Mpa, elongation −10%, MOE −110 GPa].
Matrix (No abutment) (Model C)	TRI Dental Implants Int. AG, Aargau, Switzerland	Two non-abutment implant systems—multi and bone level. Multi-level [width 3.3, 3.75, 4.1, 4.75 mm; length—6.5, 8, 10, 11.5, 13, 16 mm]; Bone level [width 3.75, 4.1, 4.7 mm; length—all as above]. Material: titanium grade 5 ELI (titanium grade 23, Ti-6Al-4V ELI). Properties: [strength UTS/Rm ≥ 900 MPa; yield strength R0.2: ≥795 MPa; elongation A: ≥10%, MOE −113.8 GPa]. 8 integrated applications [scan and smile, DHC library, Scan Bridge, TRX, Tooth in a box, LAB design, LAB milling, ACADEMY]. Design features: Matrix SlimNeck, Matrix PowerBase, Matrix SmartLock, Matrix SmartBolt. Screw heads 2.25 (Ti), 2.6 (Zi, polymers and metals) and 2.8 (zirconia and polymers) mm.
Tri (with abutment) (Model D)		Types: bone-level (TRI-Narrow, TRI-Vent) and tissue-level (TRI-Octa). Implant size: TRI-Vent and TRI-Octa [width—3.75, 4.1, 4.7 mm; length—6.5, 8, 10, 11.5, 13, 16 mm], TRI-Narrow [width—3.3 mm; length—10, 11, 13, 16 mm]. Material: titanium grade 5 ELI (titanium grade 23, Ti-6Al-4V ELI). Properties: [strength UTS/Rm ≥ 900 MPa; yield strength R0.2: ≥795 MPa; elongation A: ≥10%, MOE −113.8 GPa]. Applications: ScanBridge, Guided Surgery, Direct on MUA, Lab Design. Features: tapered press fit for abutment, internal hex, Boneadapt, sand-blasted, large-grit, double acid-etched.
Katana	Kuraray Noritake Dental, Inc., Hattersheim am Main, Germany	Composition: 9.32wt% Y_2_O_3_ 75% cubic phase. Grain size: around 4.05 μm. Flexural strength (UTML): 557MPa.

Abbreviations: mm—millimeters, ELI—extra-low interstitial (R56401), MPa—megapascals, UTS—ultimate tensile strength, R—plastic strain ratio, BLT—bone-level tapered, MOE—modulus of elasticity, GPa—gigapascals, MUA—multi-unit approach, TPC = tapered pro-conical, Zi = zirconia, CADCAM = computer-aided diagnosis, computer-aided machining.

**Table 2:** Number of elements and nodes employed for designing various models of implant systems with different specifications.

Implant System	Model Types	Crown		Implant Fixture		Abutment		Screw		Bone		Assembled	
		Nodes	Elements	Nodes	Elements	Nodes	Elements	Nodes	Elements	Nodes	Elements	Nodes	Elements
BioHorizons	(Model A)	14,776	54,030	49,229	258,472	9588	24,255	5901	9291	24,755	115,721	49,229	258,472
Straumann	(Model B)	10,617	48,315	11,739	50,259	5409	21,683	1229	4249	21,678	102,291	43,753	229,195
Matrix	(Model C)	14,192	64,989	10,437	42,696	N. A	N. A	2302	9019	25,632	122,315	48,845	261,849
Tri	(Model D)	13,754	62,258	11,658	48,704	4119	16,625	1306	4696	23,470	112,318	48,117	250,056

Abbreviation: N.A = not applicable (abutment free implant system).

**Table 3:** Material properties employed in the finite element analysis.

Component	Type	Material Composition	MOE (GPa)	Poisson’s Ratio
Implant fixture	Matrix	Grade 5 ELI	114	0.34
	Tri	Grade 5 ELI	114	0.34
	BioHorizons (TPC)	Wrought Ti-6Al-4V ELI alloy	114	0.34
	Straumann (BLT)	Roxolid	98	0.34
Abutment	Matrix	Grade 5 ELI	114	0.34
	Tri	Grade 5 ELI	114	0.34
	BioHorizons (TPC)	Wrought Ti-6Al-4V ELI alloy	114	0.34
	Straumann (BLT)	Roxolid	110	0.34
Screw	Matrix	Grade 5 ELI	114	0.34
	Tri	Grade 5 ELI	114	0.342
	BioHorizons (TPC)	Wrought Ti-6Al-4V ELI alloy	114	0.34
	Straumann (BLT)	Roxolid	110	0.34
Restorative materials	Composite	BisGMA	7	0.105
	Teflon	Polytetrafluorethylene	0.575	0.35 to 0.46
	Zirconia crown	Katana HTML	217	0.35
Tissue type	Dense trabecular bone (D2 and D3 bone)		1.37	0.3
	Cortical bone		13.7	0.3

Abbreviations: TPC—tapered pro-conical, ELI = extra-low interstitial, MOE—modulus of elasticity, GPa = gigapascals, BisGMA = bisphenol A glycidyl methacrylate, BLT = bone-level tapered, Note: Grade 5 ELI represents (titanium grade 23, Ti-6Al-4V ELI) Roxolid = 85% titanium, 15% zirconium, (UNS R56401).

**Table 4:** Von Mises stress distribution results (Mpa) for all the components for all models under static axial and oblique loading.

Loading	Model Type	Implant Fixture	Screw	Abutment	Crown	Abutment + Crown	Cortical Bone	Cancellous Bone
Static axial	Model A	45.5	13.84	45.94	25.83	45.94	20.62	4.22
	Model B	32.36	9.94	27.81	29.81	29.81	16.49	4.65
	Model C	46.3	16.85	NA	31.64	31.65	26.55	2.67
	Model D	39.18	12.89	39.18	35.57	39.17	19.78	2.61
Static oblique	Model A	201.6	47.28	220.47	99.65	220.47	60.36	7.05
	Model B	153.02	31.83	111.29	134.26	134.26	55.14	6.34
	Model C	214.06	57.46	NA	162.1	214.05	108.99	8.69
	Model D	237.83	43.33	162.13	138.44	162.13	61.33	7.93
Cyclic static	Model A	81.44	24.77	82.23	46.23	82.28	36.90	7.55
	Model B	56.43	17.33	48.49	51.98	51.97	28.75	8.11
	Model C	83.34	30.33	NA	56.95	56.95	47.80	4.81
	Model D	69.21	22.77	69.21	62.84	69.21	34.94	4.72

Note: A = BioHorizons (tapered pro-conical), B = Straumann (bone-level tapered), C = Matrix (no abutment), D = Tri (with abutment). All values are in megapascals (Mpa), NA = Not applicable.

## Data Availability

The original contributions presented in the study are included in the article, further inquiries can be directed to the corresponding author.

## References

[1] Qasim S.S., Zafar M.S., Niazi F.H., Alshahwan M., Omar H., Daood U. (2020). Functionally graded bi-omimetic biomaterials in dentistry: An evidence-based update. J. Biomater. Sci. Polym. Ed..

[2] Minocha T., Mattoo K., Rathi N. (2020). An 2/2 implant overdenture. J. Clin. Res. Dent..

[3] Pereira A.K., de Oliveira Limirio J.P., do Egito Vasconcelos B.C., Pellizzer E.P., de Moraes S.L. (2024). Mechanical behavior of titanium and zirconia abutments at the implant-abutment interface: A systematic review. J. Prosthet. Dent..

[4] Mao Z., Beuer F., Wu D., Zhu Q., Yassine J., Schwitalla A., Schmidt F. (2023). Microleakage along the implant–abutment interface: A systematic review and meta-analysis of in vitro studies. Int. J. Implant Dent..

[5] Jain S., Mattoo K., Khalid I., Baig F.A., Kota M.Z., Ishfaq M., Ibrahim M., Hassan S. (2023). A study of 42 partially edentulous patients with single-crown restorations and implants to compare bone loss between crestal and subcrestal endosseous implant placement. Med. Sci. Monit. Int. Med. J. Exp. Clin. Res..

[6] Sun Y., Shukla A., Ramachandran R.A., Kanniyappan H., Yang B., Harlow R., Campbell S.D., Thalji G., Mathew M. (2024). Fretting-corrosion at the Implant–Abutment Interface Simulating Clinically Relevant Conditions. Dent. Mater..

[7] Sasada Y., Cochran D.L. (2017). Implant-Abutment Connections: A Review of Biologic Consequences and Peri-implantitis Implications. Int. J. Oral Maxillofac. Implant..

[8] Choi S., Kang Y.S., Yeo I.S. (2023). Influence of implant–abutment connection biomechanics on biological response: A literature review on interfaces between implants and abutments of titanium and zirconia. Prosthesis.

[9] Kihara H., Hatakeyama W., Kondo H., Yamamori T., Baba K. (2022). Current complications and issues of implant superstructure. J. Oral Sci..

[10] Maeda Y., Satoh T., Sogo M. (2006). In vitro differences of stress concentrations for internal and external hex implant-abutment connections: A short communication. J. Oral Rehabil..

[11] Quaresma S.E., Cury P.R., Sendyk W.R., Sendyk C. (2008). A finite element analysis of two different dental implants: Stress distribution in the prosthesis, abutment, implant, and supporting bone. J. Oral Implantol..

[12] Elsayed M.D. (2019). Biomechanical factors that influence the bone-implant-interface. Res. Rep. Oral Maxillofac. Surg..

[13] Mitra D., Gurav P., Rodrigues S., Khobragade B., Mahajan A. (2023). Evaluation of stress distribution in and around dental implants using three different implant–abutment interfaces with platform-switched and non-platform-switched abutments: A three-dimensional finite element analysis. J. Dent. Res. Dent. Clin. Dent. Prospect..

[14] Mohamad T., Al-Adel U., Wahbeh E. (2013). The effect of dental implant length and diameter on the stress distribution at the implant-bone interface of the immediate loading implants: A 3/D finite element analysis. Al-Rafidain Dent. J..

[15] Sevimay M., Usumez A., Eskitascıoglu G. (2005). The influence of various occlusal materials on stresses transferred to implant-supported prostheses and supporting bone: A three-dimensional finite-element study. J. Biomed. Mater. Res. Part B Appl. Biomater..

[16] Wu A.Y.J., Hsu J.T., Chee W., Lin Y.T., Fuh L.J., Huang H.L. (2016). Biomechanical evaluation of one-piece and two-piece small-diameter dental implants: In-vitro experimental and three-dimensional finite element analyses. J. Formos. Med. Assoc..

[17] Cervino G., Romeo U., Lauritano F., Bramanti E., Fiorillo L., D’Amico C., Milone D., Laino L., Campolongo F., Rapisarda S. (2018). Fem and von Mises analysis of OSSTEM dental implant structural components: Evaluation of different direction dynamic loads. Open Dent. J..

[18] Shash M., Nazha H., Abbas W. (2019). Influence of different abutment designs on the biomechanical behavior of one-piece zirconia dental implants and their surrounding bone: A 3D-FEA. IRBM.

[19] Mohammadpour S., Khorramymehr S. (2020). Comparing one-and two-piece dental abutments under dynamic loading: A 3-D finite element analysis. Thesis Int. J. Eng. Technol..

[20] Barbosa F.T., Zanatta L.C.S., de SouzaRendohl E., Gehrke S.A. (2021). Comparative analysis of stress distribution in one-piece and two-piece implants with narrow and extra- narrow diameters: A finite element study. PLoS ONE.

[21] Takahashi J.M., Dayrell A.C., Consani R.L., de Arruda Nóbilo M.A., Henriques G.E., Mesquita M.F. (2015). Stress evaluation of implant-abutment connections under different loading conditions: A 3D finite element study. J. Oral Implantol..

[22] Pitta J., Hicklin S.P., Fehmer V., Boldt J., Gierthmuehlen P.C., Sailer I. (2019). Mechanical stability of zirconia meso-abutments bonded to titanium bases restored with different monolithic all-ceramic crowns. Int. J. Oral Maxillofac. Implant..

[23] Stimmelmayr M., Sagerer S., Erdelt K., Beuer F. (2013). In vitro fatigue and fracture strength testing of one-piece zirconia implant abutments and zirconia implant abutments connected to titanium cores. Int. J. Oral Maxillofac. Implant..

[24] Gao J., Min J., Chen X., Yu P., Tan X., Zhang Q., Yu H. (2021). Effects of two fretting damage modes on the dental implant–abutment interface and the generation of metal wear debris: An in vitro study. Fatigue Fract. Eng. Mater. Struct..

[25] Kaleli N., Sarac D., Külünk S., Öztürk Ö. (2018). Effect of different restorative crown and customized abutment materials on stress distribution in single implants and peripheral bone: A three-dimensional finite element analysis study. J. Prosthet. Dent..

[26] Talmazov G., Veilleux N., Abdulmajeed A., Bencharit S. (2020). Finite element analysis of a one-piece zirconia implant in anterior single tooth implant applications. PLoS ONE.

[27] Fiorillo L., Milone D., D’Andrea D., Santonocito D., Risitano G., Cervino G., Cicciù M. (2022). Finite element analysis of zirconia dental implant. Prosthesis.

[28] Deste Gökay G., Oyar P., Gökçimen G., Durkan R. (2024). Static and dynamic stress analysis of different crown materials on a titanium base abutment in an implant-supported single crown: A 3D finite element analysis. BMC Oral Health.

[29] Stawarczyk B., Keul C., Eichberger M., Figge D., Edelhoff D., Lumkemann N. (2017). Three generations of zirconia: From veneered to monolithic. Part I. Quintessence Int..

[30] TRI Dental Implants. https://tri-implants.swiss/en/matrix-line/.

[31] TRI Dental Implants. https://tri-implants.swiss/en/performance-line/.

[32] Südbeck S., Buser R., Reymus M., Hoffmann M., Edelhoff D., Stawarczyk B. (2023). A New Implant System with Directly Screwed Supraconstructions: Impact of Restoration Material and Artificial Aging on the Bending Moment. Int. J. Prosthodont..

[33] Lee J.H., Jang H.Y., Lee S.Y. (2021). Finite element analysis of dental implants with zirconia crown restorations: Conventional cement-retained vs. cementless screw-retained. Materials.

[34] Brizuela-Velasco A., Pérez-Pevida E., Jiménez-Garrudo A., Gil-Mur F.J., Manero J.M., Punset-Fuste M., Chávarri-Prado D., Diéguez-Pereira M., Monticelli F. (2017). Mechanical characterisation and biomechanical and biological behaviours of Ti-Zr binary-alloy dental implants. BioMed Res. Int..

[35] Karahalil B., Kadioglu E., Tuzuner-Oncul A.M., Cimen E., Emerce E., Kisnisci R.S. (2014). Micronucleus assay assessment of possible genotoxic effects in patients treated with titanium alloy endosseous implants or miniplates. Mutat. Res. Genet. Toxicol. Environ. Mutagen..

[36] Osman R.B., Swain M.V. (2015). A critical review of dental implant materials with an emphasis on titanium versus zirconia. Materials.

[37] Bio-Horizon Tapered Pro Conical Implant. https://www.biohorizons.com/products/TaperedProConical.

[38] Nikiel P., Wróbel M., Szczepanik S., Stępień M., Wierzbanowski K., Baczmański A. (2021). Microstructure and mechanical properties of Titanium grade 23 produced by selective laser melting. Arch. Civ. Mech. Eng..

[39] Shemtov-Yona K. (2021). Quantitative assessment of the jawbone quality classification: A meta-analysis study. PLoS ONE.

[40] Straumann Roxolid. https://www.straumann.com/en/dental-professionals/dental-implants/dental-implant-materials/roxolid.html.

[41] Boschetti E., Silva-Sousa Y.T.C., Mazzi-Chaves J.F., Leoni G.B., Versiani M.A., Pécora J.D., Saquy P.C., Sousa-Neto M.D. (2017). Micro-CT Evaluation of Root and Canal Morphology of Mandibular First Premolars with Radicular Grooves. Braz. Dent. J..

[42] Dynamic Loading Test for Endosseous Dental Implants (Shenzhen, China).

[43] Sagheb K., Görgen C.I., Döll S., Schmidtmann I., Wentaschek S. (2023). Preload and friction in an implant-abutment-screw complex including a carbon-coated titanium alloy abutment screw: An in vitro study. Int. J. Implant Dent..

[44] Mericske-Stern R., Piotti M., Sirtes G. (1996). 3-D in vivo force measurements on mandibular implants supporting overdentures. A comparative study. Clin. Oral Implant. Res..

[45] Farooq M., Sazonov E. (2016). Automatic Measurement of Chew Count and Chewing Rate during Food Intake. Electronics.

[46] Dallago M., Fontanari V., Torresani E., Leoni M., Pederzolli C., Potrich C., Benedetti M. (2018). Fatigue and biological properties of Ti-6Al-4V ELI cellular structures with variously arranged cubic cells made by selective laser melting. J. Mech. Behav. Biomed. Mater..

[47] Hjerppe J., Jung R.E., Hämmerle C.H., Özcan M., Mühlemann S. (2022). Mechanical stability of fully personalized, abutment-free zirconia implant crowns on a novel implant-crown interface. J. Dent..

[48] Fabbri G., Fradeani M., Dellificorelli G., De Lorenzi M., Zarone F., Sorrentino R. (2017). Clinical Evaluation of the Influence of Connection Type and Restoration Height on the Reliability of Zirconia Abutments: A Retrospective Study on 965 Abutments with a Mean 6-Year Follow-Up. Int. J. Periodontics Restor. Dent..

[49] Yang F., Liu D., Yin W., Yuan C., Hu Y., Xu J., Yang Y., Tang J., Chen J. (2024). Three-dimensional finite element analysis of the biomechanical behaviour of different dental implants under immediate loading during three masticatory cycles. Heliyon.

[50] Lee H., Jo M., Sailer I., Noh G. (2022). Effects of implant diameter, implant-abutment connection type, and bone density on the biomechanical stability of implant components and bone: A finite element analysis study. J Prosthet Dent..

[51] Wittneben J.G., Joda T., Weber H.P., Brägger U. (2017). Screw retained vs. cement retained implant-supported fixed dental prosthesis. Periodontology 2000.

[52] Cho S.M., Byun S.H., Ahn S.Y., Han H.S., On S.W., Park S.Y., Yi S.M., Park I.Y., Yang B.E., Kim L.K. (2025). Biomechanical Evaluation of a Novel Non-Engaging Abutment and Screw in Internal Implant Systems: Comparative Fatigue and Load Testing. J. Funct. Biomater..

[53] Arun Kumar G., Mahesh B., George D. (2013). Three dimensional finite element analysis of stress distribution around implant with straight and angled abutments in different bone qualities. J. Indian Prosthodont. Soc..

[54] Poovarodom P., Rungsiyakull C., Suriyawanakul J., Li Q., Sasaki K., Yoda N., Rungsiyakull P. (2023). Effect of implant placement depth on bone remodeling on implant-supported single zirconia abutment crown: A 3D finite element study. J. Prosthodont. Res..

[55] Geramizadeh M., Katoozian H., Amid R., Kadkhodazadeh M. (2017). Finite element analysis of dental implants with and without microthreads under static and dynamic loading. J. Long. Term. Eff. Med. Implant..

[56] Pirmoradian M., Naeeni H.A., Firouzbakht M., Toghraie D., Khabaz M.K., Darabi R. (2020). Finite element analysis and experimental evaluation on stress distribution and sensitivity of dental implants to assess optimum length and thread pitch. Comput. Methods Programs Biomed..

[57] Dan Z., Lu J., Chang H., Qu P., Zhang A., Fang Z., Dong Y., Wang Y., Zhou L. (2020). High-Stress Compressive Creep Behavior of Ti-6Al-4V ELI Alloys with Different Microstructures. MATEC Web of Conferences.

[58] Amin Zarei M., Shabestari M.G., Shabestari S.G., Abedi H. (2025). Microstructural Heterogeneity and Anisotropic Mechanical Properties of Titanium alloys manufactured by Wire Arc Additive Manufacturing: A review. J. Mater. Res. Technol..

[59] Tshephe T.S., Akinwamide S.O., Olevsky E., Olubambi P.A. (2022). Additive manufacturing of titanium-based alloys-A review of methods, properties, challenges, and prospects. Heliyon.

[60] Jawed S.F., Rabadia C.D., Khan M.A., Khan S.J. (2022). Effect of alloying elements on the compressive mechanical properties of biomedical titanium alloys: A systematic review. ACS Omega.

[61] Bayata F., Yildiz C. (2020). The effects of design parameters on mechanical failure of Ti-6Al-4V implants using finite element analysis. Eng. Fail. Anal..

[62] Mattoo K., Garg R., Bansal V. (2014). Designing the occlusion for a single tooth implant in a compromised occlusion. J. Med. Sci. Clin. Res..

[63] Kumar L., Verma A., Pal U.S., Mattoo K., Algarni Y.A., Bin Hassan S.A., Baba S.M., Jeri S.Y., Khateeb S.U. (2023). Influence of prosthodontic rehabilitation using zygomatic implants in Covid 19 related mucormycosis (rhino–orbital–cerebral) maxillectomy patients upon post-operative stress, anxiety and functional impairment: A prospective cohort study. Clin. Interv. Aging.

[64] Sindi A.S., Kumar L., Verma A., Pal U.S., Sayed M.E., Mattoo K., Shafi S. (2023). Prosthodontic rehabilitation’s role in alleviating anxiety and depression in mucormycosis-induced maxillectomy patients post-COVID-19. Med. Sci. Monit..

